# Inferring the natural history of HPV from global cancer registries: insights from a multi-country calibration

**DOI:** 10.1038/s41598-024-65842-3

**Published:** 2024-07-10

**Authors:** Robyn M. Stuart, Jamie A. Cohen, Romesh G. Abeysuriya, Paula Sanz-Leon, Cliff C. Kerr, Darcy Rao, Daniel J. Klein

**Affiliations:** 1 Gender Equality Division, Bill & Melinda Gates Foundation, Seattle, WA USA; 2 Global Health Division, Institute for Disease Modeling, Bill & Melinda Gates Foundation, Seattle, WA USA; 3 Burnet Institute, Melbourne, VIC Australia; 4 Brain Modelling Group, QIMR Berghofer Medical Research Institute, Brisbane, QLD Australia

**Keywords:** Software, Viral infection, Computational models

## Abstract

Human papillomavirus (HPV) is the cause of almost all cases of cervical cancer, a disease that kills some 340,000 women per year. The timeline from initial infection with HPV to the onset of invasive cervical cancer spans decades, and observational studies of this process are limited to settings in which treatment of precancerous lesions was withheld or inadequate. Such studies have been critical for understanding the natural history of HPV. Modeling can shed additional insight on the natural history of HPV, especially across geographical settings with varying prevalence of factors known to affect the host-side immune response to HPV, such as HIV and tobacco use. In this study, we create models for the 30 most populous countries in Sub-Saharan Africa, each with country-specific demographic, and behavioral inputs. We found that it was not possible to fit the data if we assumed that the natural history parameters were exactly identical for all countries, even after accounting for demographic and behavioral differences, but that we could achieve a good fit with the addition of a single immunocompetence parameter for each country. Our results indicate that variation in host immune responses may play a role in explaining the differences in the burden of cervical cancer between countries, which in turn implies a greater need for more geographically diverse data collection to understand the natural history of HPV.

## Introduction

Human papillomavirus (HPV) is an extremely common sexually transmitted infection, with an estimated lifetime incidence of around 80% in the United States^1^. More than 90% of infections clear on their own within 2 years, but in some cases high-risk HPV infection persists, leading to pre-cancerous lesions which may progress to invasive cancer^2,3^. HPV has been implicated as the causative agent in more than 90% of anal and cervical cancers, about 70% of vaginal and vulvar cancers, and more than 60% of penile cancers^4^. Of these, cervical cancer forms the largest burden, with more than 600,000 new cases and 342,000 deaths estimated in 2020^5^, making it the fourth most common cancer among women globally.

While the association between HPV and cervical cancer is well-established, the probability of, and time taken for, progression from HPV infection through stages of cervical intraepithelial neoplasia (CIN) and to invasive cervical cancer is unobserved. Two broad categories of natural history estimates exist: panel-based estimates and model-driven estimates. Panel-based estimates typically come from settings in which treatment of precancerous lesions was withheld for some reason^6^. Model-driven estimates, on the other hand, are generated when models of HPV and cervical disease are calibrated to available data, such as HPV prevalence, the age distribution of cervical cancer, and sexual behavior data^7–10^.

Model-driven estimates of the natural history of HPV overcome several of the challenges of observational studies, e.g. they are much cheaper and simpler to conduct. However, they also have several drawbacks. Chief among these is an identifiability problem. Models of HPV and cervical disease often contain hundreds of parameters, while the dimensionality of the underlying data is much smaller. As a result, there are infinitely many parameter combinations that would replicate the observed data. In order to make it feasible to fit such a model to data, a decision must be made as to which parameters to vary. Demographic, behavioral, and programmatic parameters are clearly context-dependent, but it may be reasonable to assume natural history is not, i.e. that a woman’s chances of either clearing an infection or progressing to invasive cancer (and the timelines for doing so) do not vary from country to country. Both within HPV and beyond, this is a common assumption when fitting disease models across multiple settings^10–12^. Whether or not it is a reasonable assumption depends to a large extent on whether the model is able to capture the variations in factors that may alter a host’s immune response to infection, such as age, comorbidities, prior immune history, and other confounders. HIV-induced immunosuppression is especially influential on a host’s ability to clear HPV infection^13^.

The goal of this paper is to determine how much of the variation in the distribution of cervical cancer burden across 30 countries in Sub-Saharan Africa can be explained by differences in demographic, programmatic, and behavioral factors alone, and how much would appear to be attributable to underlying differences in host immune responses and thus reflected in different natural history parameters. A priori, we expect that if we use a model that does not account for HIV, the natural history will vary from country to country, since HIV is known to have such a substantial effect on HPV clearance and progression rates^13^. A secondary goal is to determine the minimal number of parameters that need to be varied in order to capture the observed between-country differences in cervical cancer burden. Generally speaking, model fits can always be improved by adding more parameters, but at the expense of parsimony and generalizability. We provide an example illustrating how models that do not constrain some aspects of the disease’s natural history may lead to overfitting, especially when these models are based on very limited quantities of publicly available data.

## Methods

### Country scope

For our study, we include the 30 most populous countries in Sub-Saharan Africa: Nigeria, Ethiopia, Democratic Republic of the Congo (DRC), Tanzania, South Africa, Kenya, Uganda, Angola, Mozambique, Ghana, Madagascar, Cameroon, Côte d'Ivoire, Niger, Burkina Faso, Mali, Malawi, Zambia, Senegal, Chad, Somalia, Zimbabwe, Guinea, Rwanda, Benin, Burundi, South Sudan, Togo, Sierra Leone, and the Republic of Congo. Collectively, these countries comprise 1.1 billion people and experienced ~100,000 cases of cervical cancer in 2020, which equates to ~ 95% of the population and 90% of the estimated total burden of cervical cancer within Sub-Saharan Africa. The life expectancy at birth ranges from 53 (Nigeria) to 68 (Senegal), and HIV prevalence among women aged 15–49 ranges from 0.1% (Somalia) to 24.5% (South Africa). The burden of cervical cancer also varies significantly across these countries, with age-standardized incidence rates (ASIR) per 100,000 women ranging from 10.4 in Niger to 67.9 in Malawi (GLOBOCAN, 2020). Table S1 summarizes these and other key features of the modeled countries. Since our study is a simulation study using publicly available data, informed consent was not required.

### Model framework and timeframe

We use HPVsim^14,15^, a flexible software package that we developed for creating agent-based models of HPV transmission and cervical disease progression. A model created using HPVsim consists of a population of agents who interact with each other through sexual networks, over which multiple co-circulating HPV genotypes can be transmitted. Persistent HPV infections can progress to cervical dysplasia, and subsequently regress spontaneously or proceed to invasive cervical cancer. HPVsim can also capture the effects of interventions, including vaccination, screening, and treatment. Using HPVsim, we create models for each of the 30 countries over the period 1960–2020. In the following sections we provide more details on the specific components of the framework and how they have been customized to create models for this study.

### Modeling populations and networks

HPVsim includes default values for all of the parameters needed to create a population of agents and the sexual networks that connect them. However, many of these parameters must be overwritten with context-specific values in order to create a model for a given country. Location-specific demographic parameters for each of the 30 countries are included as part of the standard installation of the HPVsim package, with crude birth rates by year from the World Bank^17^, age- and sex-specific mortality rates from the UN, and overall population size from the WPP’s mid-fertility projections^18^. These demographic data allow us to simulate populations of agents over time, which we can then validate against data on population age pyramids (Figure S1). We then customize each country’s sexual network parameters. Specifically, we use the DHS Statcompiler^19^ to extract aggregated survey data on the age of sexual debut, the proportion of the population who report being married or cohabiting, the proportion of the population who report sex with a non-marital partner, and reported age differences between partners. For each country, we adjust the mean degree distribution of the sexual network for both males and females, and partner age differences. Further parameters are adjusted during the model calibrations, with more details contained in the section on the calibration approach. Figures S2–S6 illustrate the models’ fidelity to these sexual behavior data.

### Modeling HPV transmission

For this study, we simulate 4 groups of HPV genotypes. HPV16 and HPV18 are each modeled individually, and in addition we include a pooled group of high-risk types included in the nonavalent vaccine (31, 33, 45, 52, and 58) which we term “Hi5”, and a pooled collection of other high-risk oncogenic types (35, 39, 51, 56, and 59) which we term “OHR”.

### Modeling interventions

Prophylactic vaccination, cervical screening, treatment of precancerous lesions, and treatment of cancer are all established interventions that aim to reduce the burden of HPV and cervical disease. We searched for data on coverage levels of all of these interventions for our models.

The proportion of women aged 30–49 in Sub-Saharan Africa who had ever received a cervical screen has been estimated at 15%^20–22^. Across the four countries that had published survey data on precancer treatment, coverage was estimated at around 80%^21^. Substantial variation in both the coverage levels and the quality of screening programs has been reported. In light of the fact that data availability is so inconsistent, we make the decision not to include any screening or treatment effects in our models. This means that, for any country in which screening and treatment have been effectively operating at scale, we will overestimate the burden of cancers, since some of these cancers would not have occurred if they had been detected and removed early.

Several countries within Sub-Saharan Africa had initiated routine HPV vaccination programs by 2020, including Côte d’Ivoire, Ethiopia, Kenya, Malawi, Rwanda, Senegal, South Africa, Tanzania, Uganda, Zambia, and Zimbabwe^23^. Overall coverage within the countries with vaccination programs was estimated at 20%. Given that these vaccination programs were introduced relatively recently, it will not affect our estimates of cervical disease over the study period (1960–2020), and for simplicity we do not include these programs in our models. This is consistent with the approach taken in other large modeling studies^24^.

### Modeling disease progression

Because we specifically wish to understand the degree to which HPV’s natural history might vary from one country to another, our natural history model must be constructed using parameters that can be informed by country-specific data and not rely on estimates derived from other contexts. The most comprehensive source of cervical cancer data is GLOBOCAN, which publishes IARC-certified cancer registry data where available and produces estimates for countries without registry data^25,26^. Data on HPV prevalence are also collated by the ICO/IARC Information Centre on HPV and Cancer. Table 1 summarizes data availability by country.

**Table 1:** Data availability from GLOBOCAN^25,26^ and the ICO/IARC Information Centre on HPV and Cancer.

Data type	Available for
Number of cases of cervical cancer by age (5-year age bins, 15–85+), 2020	All 30 countries
Age-specific rates of cervical cancer incidence and mortality, 2020	All 30 countries
Distribution of HPV genotypes among women with invasive cervical cancer (samples sizes in parentheses)	Ethiopia (291), Guinea (44), Kenya (233), Mali (157), Mozambique (292), Nigeria (145), Senegal (199), South Africa (674), Tanzania (97), Uganda (321), Zimbabwe (95)

In the settings that we are modeling, data on the rate of progression of precancerous lesions are scarce, mostly because cervical screening programs are not operating at large scales. As a result, we cannot produce reliable, context-specific, data-driven estimates of the probabilities of progressing from productive infection to low- or high-grade lesions within a given timeframe. We can, however, propose a simple yet biologically-motivated model for the probability of developing cervical cancer over time. Given that we are modeling a binary outcome (cancer/no cancer) over time which is known to depend on the extent and persistence of dysplasia in the cervical cells, we can adopt a standard geometric distribution to approximate the probability of cancer developing over time, i.e.:

1 $$prob(cancer\; begins\; T\; years \;after \;infection|p,\; dys{p}_{T,g}) = 1-(1-{p}_{g}{)}^{dys{p}_{T,g}}$$

Here, *p*_*g*_ can be considered as the (time-constant) probability of a given dysplastic cell becoming cancerous at any point in time, while the exponent *dysp*_*T,g*_ represents the cumulative dysplastic cell time (i.e., the sum, at time *T*, of the number of years each cell has been infected with genotype *g*). We then need a way to approximate *dysp*_*T,g*_*.* Before proposing a model for this, we start by considering the general properties that it should have. We know the number of cells with a non-zero probability of becoming cancerous should be close to zero for the first few years of infection to allow time for precancerous lesions to develop, and that as *T* increases, *dysp*_*T,g*_ should be monotonically increasing. A simple ramp function, similar to that depicted in Schiffman et al.^27^, meets these requirements. We use a smooth approximation of a ramp function, namely a modified softplus function, to approximate the lateral expansion of cells with a non-zero probability of becoming cancerous at *T* years:

2 $${dysp}_{T,g} = \frac{2log(exp({k}_{g}T)+1)}{{k}_{g}}-\frac{2\text{log}\left(2\right)}{{k}_{g}}-T.$$

Here, *k*_*g*_ is an ‘aggressiveness’ parameter, which varies by genotype and describes how quickly the infection progresses to a potentially oncogenic state within an individual woman. The softplus function described in Eq. 2 has the convenient property that its derivative is a logistic function. This means that our approximation of the growth rate of cells with oncogenic potential can also be considered as the cumulative severity of a lesion whose lateral expansion evolves over time according to:

3 $${dysp}_{t,g} = \frac{2}{1+exp(-{k}_{g}t)}-1, t\in [0,T]$$

Given that the depth of a lesion is frequently taken as one of the proxy markers for clinical severity, this model of lesion depth is consistent with lesions that progress relatively quickly at first, and then more slowly.

Our model so far does not account for individual variations in women’s immunity, which cause cervical disease to progress faster/slower in some individuals than others. We therefore incorporate an “immunocompetence” parameter, which does not vary by genotype but does vary between individuals. We will assume that this acts as a scale factor on the k_g_ terms in Eqs. 2–3. Initially, we will assume that within a given setting, this immunocompetence parameter, which we refer to as *relsev*_*i*_ for individuals i = 1…N in a population, is drawn from a normal distribution, i.e.:

4 $$relse{v}_{i,c}\sim N({\nu }_{c}, 0.1)$$

where the mean ν_c_ varies by country c. We set ν_c_ to 1 by default, but we estimate it during calibrations later (see section below).

In order to generate the durations of infection (*T*) for each woman, we draw samples from a log-normal distribution whose mean and variance depend on the infecting genotype and are determined by fitting to the expected clearance rates of infection over time^3^. At the point of infection, we calculate the probabilities given in Eq. 1 and apply them to determine the women who acquire cancer. The remaining women are then scheduled to clear their infections at the conclusion of the duration they were initially assigned.

All in all, our natural history model consists of three unknown parameters: *p*_*g*_, *k*_*g*_, and ν_c_. Because Eq. 1 is overdetermined, we fix *p*_*g*_ and estimate *k*_*g*_.

### Calibration approach

We carry out three separate calibrations, differentiated by the number of natural history parameters that we allow to vary. Firstly, we run a constrained calibration in which a single set of the four *k*_*g*_ parameters (one per genotype) is estimated for all 30 countries. Secondly, we run an ‘immuno-varying’ calibration, in which we fix the *k*_*g*_ parameters to be the same for all 30 countries, but this time allow the mean relative severity (v_c_ in Eq. 4) to vary, implying 30 parameters instead of 4 for the natural history component. Finally, we run an unconstrained calibration in which the natural history parameters are estimated separately for each country. In all three calibrations, we vary 4 sexual behavior parameters per country: the proportion of the population who have extra-marital relationships (prior for males: U(0.1, 0.7), prior for females: U(0.05,0.5)) and the proportion of the population who participate in casual relationships when not married (prior for males: U(0.1, 0.6), prior for females: U(0.01,0.6)). Our prior distributions for HPV16 and HPV18 are k_g_ ~ U(0.25,0.45), and for Hi5 and OHR we use k_g_ ~ U(0.15, 0.4). A given parameter set consists of a single value for each of these parameters, and will produce a given set of model outputs. We calculate the overall mismatch produced by a given parameter set as the sum of the normalized absolute differences between the outputs of the model using that parameter set and the data listed in Table 2.

**Table 2:** Calibration methods and the number of parameters varied for each.

Calibration	Description	Parameters calibrated
Constrained	A common set of natural history parameters is estimated for all countries	Common across countries: 4 natural history parameters k_g_; total parameters = 4 Unique to each country: 4 sexual behavior parameters per country; total unique parameters = 120 Total: 124
Immuno-varying	A common set of natural history parameters is fixed for all countries, but the mean immunocompetence varies from country to country	Common across countries: None (natural history (k_g_) fixed at prior means) Unique parameters: 1 natural history parameter per country (ν_c_, the mean of the relsev distribution, estimated from a prior distribution of U(0.5, 1.5)) plus 4 sexual behavior parameters per country; total unique parameters = 150 Total: 150
Unconstrained	Natural history parameters free to vary from country to country	Common: None Unique: 4 natural history parameters (k_g_) for each country plus 4 sexual behavior parameters per country; total unique parameters = 240 Total: 240

We use a hyperparameter optimization algorithm, as implemented in the Optuna package^28^, to search the parameter space using a tree-structured Parzen estimator, to find the parameter sets that minimize the overall mismatch. For the immuno-varying and constrained calibrations, we run 20,000 trials and calculate an overall mismatch as the sum of the mismatches for each country. For the unconstrained calibration, we run 3000 trials per country.

## Results

**Figure 1:**
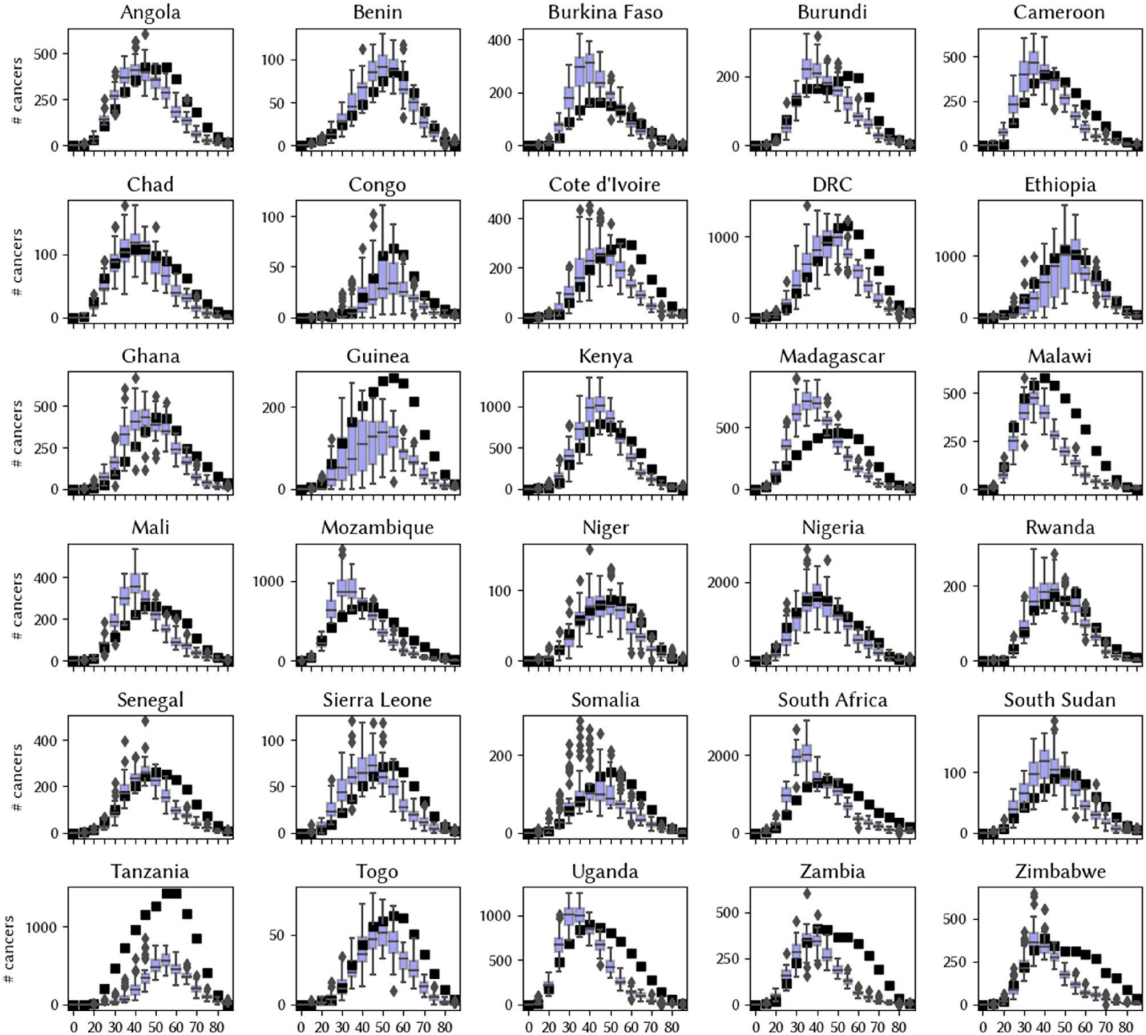
Model estimates generated from the top 50 best-fitting parameter sets (out of 20,000 trials) from the constrained calibrations (box plots) along with data from GLOBOCAN on the distribution of cervical cancer by age.

**Figure 2:**
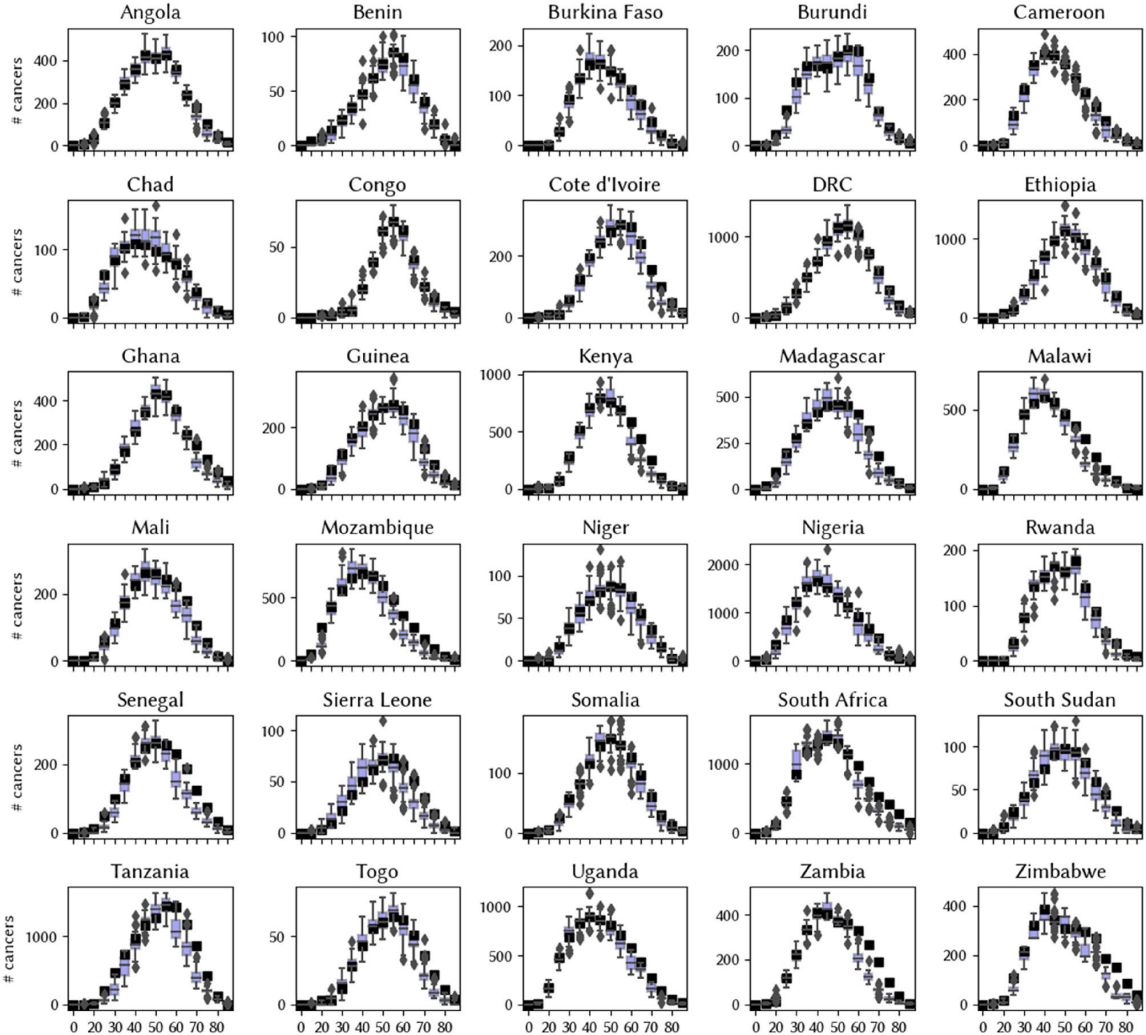
Model estimates generated from the top 50 best-fitting parameter sets (out of 20,000 trials) from the immuno-varying calibrations (box plots) along with data from GLOBOCAN on the distribution of cervical cancer by age.

We find that we cannot achieve a good fit if we constrain all the natural history parameters to be the same for all countries (Fig. 1), even though we have accounted for demographic and behavioral differences within the models. However, with our immuno-varying calibration, we find that the addition of a single immunocompetence parameter for each country means that we are able to achieve a reasonably good fit to the data on cervical cancer estimates by age (Fig. 2). Analogous results from our third (unconstrained) calibration are shown in the Supplementary Materials—we find that we can easily fit the available data on cervical cancer estimates by age if we allow the natural history parameters to vary from country to country (Figure S7). For all three calibrations, we also obtain a good fit to the distribution of HPV types found within women with invasive cervical cancer (Figures S8–S10).

**Figure 3:**
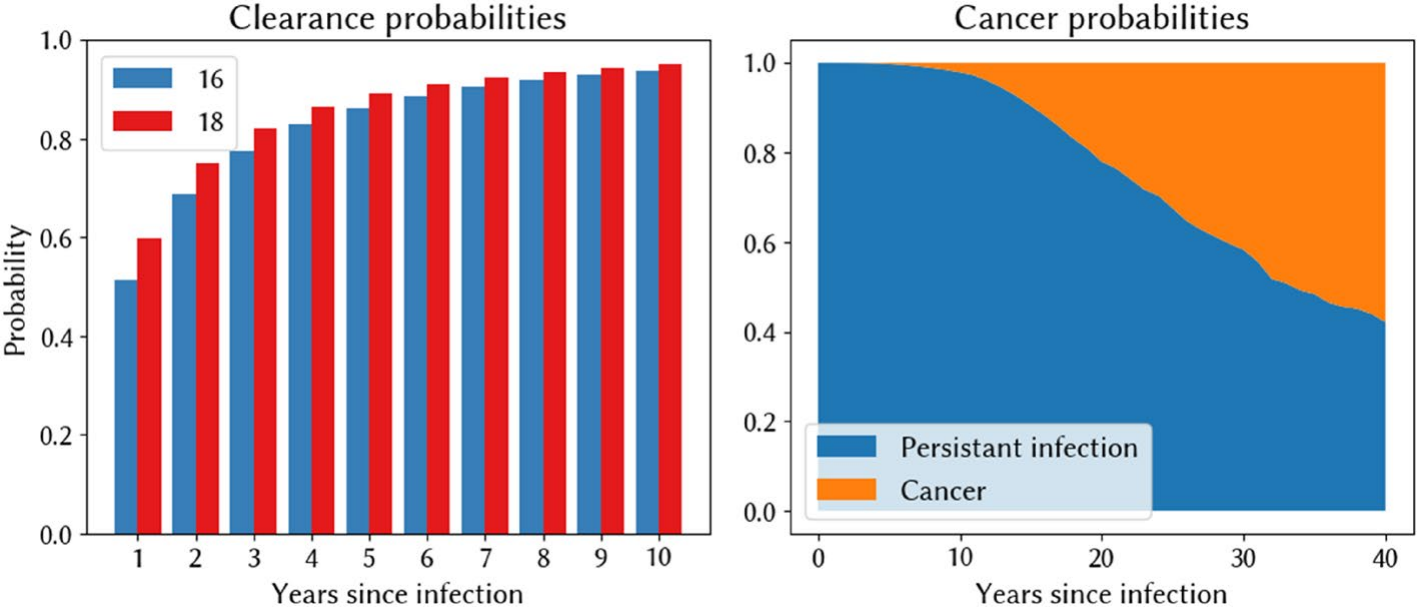
Implied probabilities of clearing infection for HPV 16 and 18 and the share of all infections that advance to cancer, estimated using the best-fitting parameter set from the immuno-varying model.

In Fig. 3, we show the estimated probabilities of clearance and invasion by infection duration, as derived from the best-fitting parameter sets from the immuno-varying calibration. Consistent with estimates from throughout the literature, our natural history model implies that 80–90% of infections clear within 2 years, with < 5% remaining after 10 years. The probability of cancer developing increases with duration of infection.

**Figure 4:**
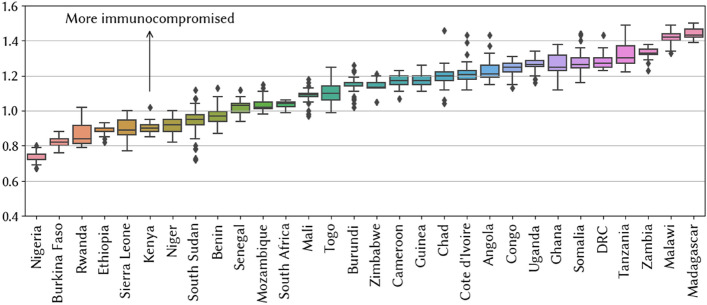
Mean estimated level of immunocompromise for individuals in each country. Box plots correspond to estimates from the 50 best-fitting parameter sets.

Our immuno-varying model results in an estimate of a mean immunocompetence parameter (ν_c_ in Eq. 4), which modifies the relative immune profiles for individuals in each country. Figure 4 shows the estimates we obtain from the 50 best-fitting parameter sets for each country. Values greater than 1 suggest that individuals are more at risk of severe disease outcomes.

## Discussion

We found that our model was unable to explain the differences in the data on the age distribution of cervical cancer cases via demographic and behavioral differences alone. Allowing the natural history parameters to vary across countries meant that we could get a good fit (results shown in Supplementary Materials), but at the cost of having more than 200 parameters and no cohesive insight into HPV's natural history. We found that the inclusion of an immunocompetence parameter helped strike a balance between flexibility and tractability.

The results that we presented in this work have a number of possible interpretations. One interpretation is that there is insufficient high-quality data to be able to produce model-driven estimates of the natural history of HPV in many countries. Although we were able to develop a data-driven model for progressing from HPV infection to cervical cancer, we were not able to infer rates of progression through clinical stages of precancer, which would be essential knowledge for designing optimal screening programs and predicting or evaluating the impact of other interventions, such as catch-up vaccination campaigns or novel therapeutics. In the absence of country-specific data, a number of other published modeling exercises have used estimates of the rates of progression from data-rich settings in order to model data-scarce settings^24^. While this approach ignores host-side factors that could result in differences from country to country, it avoids the need to independently fit the natural history parameters on the basis of very scant data, which may produce misleading results. For example, the ‘best-fitting’ natural history parameters for Tanzania and Uganda from the unconstrained calibration imply that women in these two countries would have different risks of cancer—a conclusion that is difficult to justify in the absence of a compelling underlying reason.

Another possible interpretation of our analyses is that there are evidently many factors that lead to differences in health outcomes between countries that are not explicitly captured by mathematical disease models. Here, we endeavored to capture differences in demographic and behavioral factors, and asked what remaining differences appeared in the data that might be attributable to differences in the natural history. Residual differences may often arise due to unmodeled differences in health systems or programming. In addition to screening coverage, the quality of treatment, rates of loss to follow-up, and the sensitivity of the diagnostic (which may vary considerably between individual practitioners) are difficult to observe but influential factors. Moreover, there are many differences in sexual behavior that are not captured in DHS surveys, and differences in general knowledge about HPV and cervical disease. For a model to be truly representative of the population that it simulates, it would ideally be constructed by modelers who were close enough to the data and local context to understand how to adjust model inputs to account for these factors.

Our study focused on variations in the age-specific distributions of cervical cancer cases as reported by IARC. A related study in this vein used IARC’s HPV prevalence surveys, reported for a subset of countries, and identified clear differences in the age-specific curves of HPV prevalence^29^. In high-income countries, HPV prevalence typically peaks shortly after sexual debut and declines in older age groups, but in several Sub-Saharan African countries, including South Africa, Tanzania, and Senegal, HPV prevalence is reported to remain high in middle and older aged women. Whether this can be attributed to differences in incidence patterns arising from variations in sexual behavior or to differences in rates of persistence of prevalent infections is not immediately clear. An observational study on a similar phenomenon in Costa Rica suggested a role for both^30^. Cohort effects could also be at play. In our work, we attempted to control for differences in sexual behavior, and found that differences in persistence may explain the residual differences.

We have already alluded to some of the limitations of this work, in particular the data constraints. We also note the limitations of the modeling, which (like all models) is only a rough description of reality. This paper represents the first published application of the HPVsim modeling software to create country-specific models, and we made many improvements to the software in the process of conducting these analyses. We expect that HPVsim will continue to be iteratively refined over the course of future applications. In particular, our models here did not capture the influence of HIV, known to be an important factor for HPV’s natural history. We intend to include this in follow-up work, but omitting it here (as well as other known confounders, like tobacco use) meant we could interrogate the possible differences in HPV’s natural history without specifically ascribing them to any one cause.

Our analyses raise questions about the best way to conduct multi-country modeling exercises, specifically which factors to vary and which to assume constant. The immuno-varying calibration approach that we took derives from similar hierarchical statistical frameworks, including mixed-effect models and Bayesian hierarchical models, which aim to capture the differences between groups that may still bear overall similarities. If the natural history parameters are assumed not to change, then differences in country-level outcomes must be ascribed to behavioral and programmatic or health-system differences, many of which may be difficult to capture within mathematical models. On the other hand, allowing variation in the natural history parameters may imply unjustifiable differences in host-level factors. In any modeling study, it is essential to understand what drives country-level differences in cervical disease outcomes, especially when this is so influential on intervention impact. A balanced approach, that carefully considers and incorporates sources of variability, is the most robust solution.

### Supplementary Information

Supplementary Information.

## Data Availability

The datasets used in this study are all available from GLOBOCAN (see references in Table 1). The HPVsim code is available at hpvsim.org, and all the scripts used for the analyses in this paper are available at github.com/amath-idm/hpvsim_multical. This repository also includes all the data that were used in the analyses within the ‘data’ subfolder.
