# Supplementary material for: Inferring the natural history of HPV from global cancer registries: insights from a multi-country calibration
Source: Sci Rep. 2024 Jul 10;14:15875. doi: 10.1038/s41598-024-65842-3 (PMC11233645; doi:10.1038/s41598-024-65842-3)
Supplement: Supplementary file 1 — Supplementary Information. [file 41598_2024_65842_MOESM1_ESM.docx]

**Inferring the natural history of HPV from global cancer registries: supplementary materials**

[Table S1. Country scope 2](#_Toc150870107)

[Figure S1. Population pyramids 3](#_Toc150870108)

[Figure S2. Share of females who ever had sex by age 4](#_Toc150870109)

[Figure S3. Sexual mixing matrices 5](#_Toc150870110)

[Figure S4. Age differences between partners 6](#_Toc150870111)

[Figure S5. Share of females married by age 7](#_Toc150870112)

[Figure S6. Females with 1+ casual partner by age 9](#_Toc150870113)

[Figure S7. Fit to cancers by age from unconstrained calibrations 10](#_Toc150870114)

[Figure S8. Fit to type distribution among invasive cervical cancers - constrained calibration 11](#_Toc150870115)

[Figure S9. Fit to type distribution among invasive cervical cancers - immunovarying calibration 12](#_Toc150870116)

[Figure S10. Fit to type distribution among invasive cervical cancers - unconstrained calibration 13](#_Toc150870117)

[Figure S11. Posterior distributions – constrained calibration 14](#_Toc150870118)

[Figure S12. Posterior distributions – unconstrained calibration 14](#_Toc150870119)

[References 15](#_Toc150870120)

#

# Table S1. Country scope

| **Country** | **Cancers** | **Population size** | **ASIR 2020** | **Life expectancy** | **HIV prevalence (F15-49)** |
| --- | --- | --- | --- | --- | --- |
| Nigeria | 11787 | 218,625,385 | 18.4 | 53 | 1.80% |
| Ethiopia | 7445 | 121,922,416 | 21.5 | 65 | 1.10% |
| DRC | 7772 | 96,511,548 | 31.9 | 60 | 0.90% |
| Tanzania | 5234 | 63,931,876 | 62.5 | 66 | 5.70% |
| South Africa | 10698 | 61,081,076 | 35.3 | 65 | 24.50% |
| Kenya | 5234 | 56,645,588 | 31.3 | 63 | 5.40% |
| Uganda | 6959 | 49,333,704 | 56.2 | 63 | 6.60% |
| Angola | 3195 | 35,405,823 | 37.6 | 62 | 2.10% |
| Mozambique | 5313 | 33,413,232 | 50.2 | 61 | 10% |
| Ghana | 2797 | 32,642,373 | 27.4 | 64 | 2.40% |
| Madagascar | 3760 | 29,434,823 | 41.2 | 65 | 0.40% |
| Cameroon | 2770 | 28,161,144 | 33.7 | 61 | 3.90% |
| Côte d'Ivoire | 2067 | 27,974,485 | 31.2 | 59 | 2.70% |
| Niger | 622 | 26,412,604 | 10.4 | 61 | 0.20% |
| Burkina Faso | 1132 | 22,313,534 | 18.2 | 60 | 0.80% |
| Mali | 1932 | 21,691,072 | 36.4 | 59 | 0.90% |
| Malawi | 4145 | 20,341,255 | 67.9 | 64 | 9.40% |
| Zambia | 3161 | 19,652,620 | 65.5 | 62 | 13.80% |
| Senegal | 1937 | 17,826,571 | 36.3 | 68 | 0.40% |
| Chad | 890 | 17,589,364 | 20.2 | 53 | 1.30% |
| Somalia | 1055 | 16,985,528 | 25.1 | 56 | 0.10% |
| Zimbabwe | 3043 | 15,378,243 | 61.7 | 61 | 14.40% |
| Guinea | 2068 | 14,008,576 | 50.1 | 59 | 2% |
| Rwanda | 1229 | 13,736,863 | 28.2 | 67 | 3% |
| Benin | 560 | 12,902,012 | 15.1 | 60 | 1.10% |
| Burundi | 1581 | 12,767,455 | 49.3 | 62 | 1.20% |
| South Sudan | 711 | 11,504,110 | 20.5 | 55 | 2.60% |
| Togo | 455 | 8,751,274 | 19.1 | 61 | 2.50% |
| Sierra Leone | 504 | 8,368,970 | 21.2 | 60 | 1.80% |
| Congo | 350 | 5,849,868 | 22.4 | 64 | 5.60% |

**Caption**: key features of the modeled countries. ASIR: Age-standardized incidence rate

# Figure S1. Population pyramids


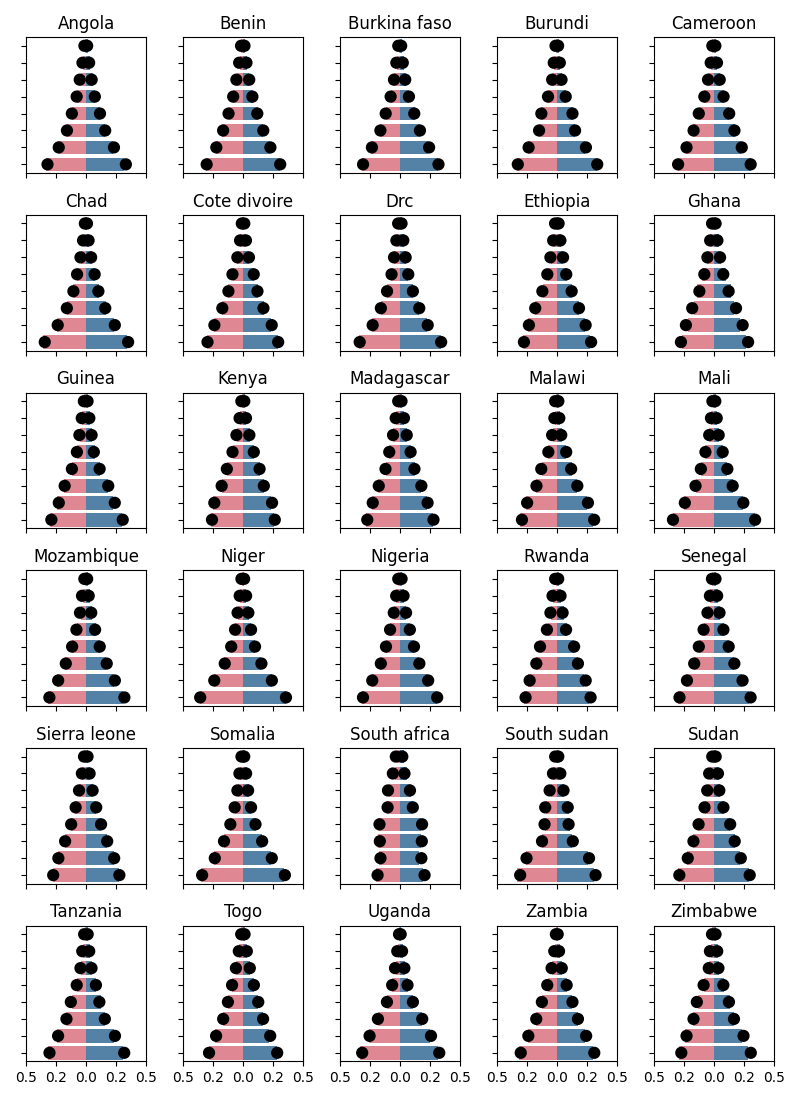


**Caption**: Model-simulated shares of females (pink) and males (blue) in each age bracket in 2020, alongside data from the UN WPP (black dots).

# Figure S2. Share of females who ever had sex by age


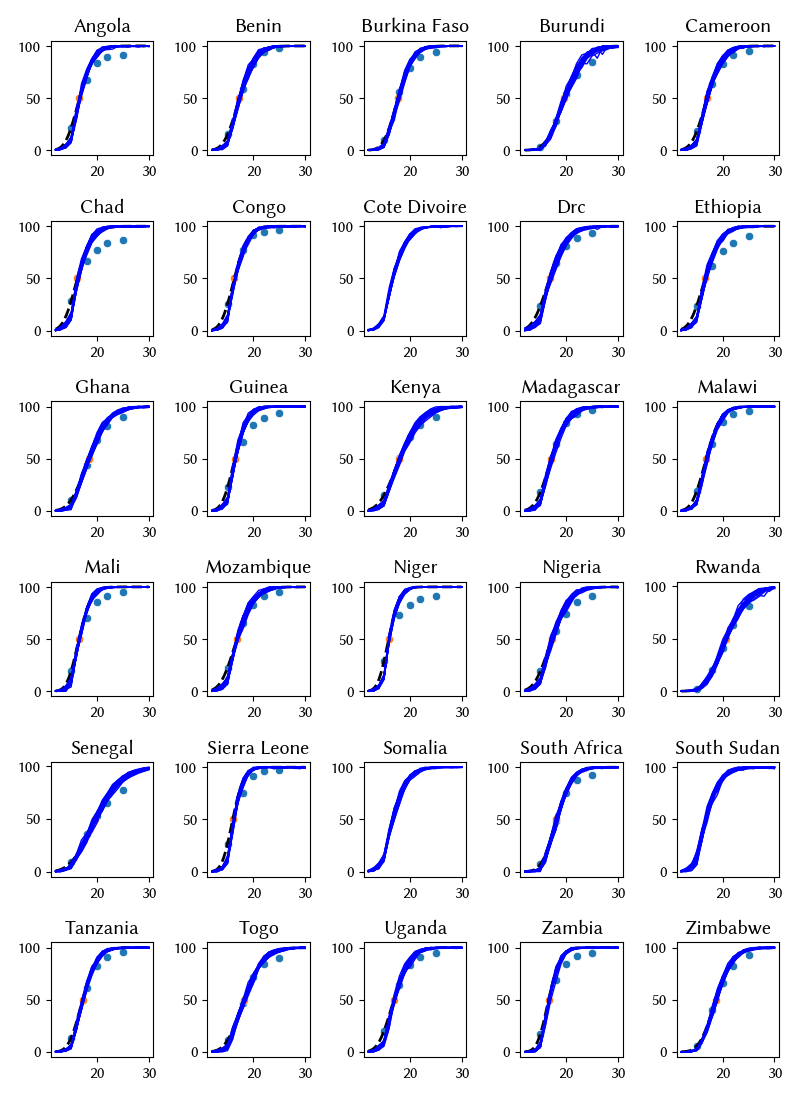


**Caption**: For each country, we collate data from DHS surveys (see sources below) on the proportion of girls/women who report ever having had sex by age 15, 18, 20, 22, and 25 (blue dots) and the median age at first sex (orange dots). We then treat these data points as quantiles of a lognormal distribution, and estimate the mean and standard deviation for each, which gives us an inferred distribution of the age of sexual debut (black dotted lines). Finally, we input these inferred distributions into the model for each country, and output the simulated proportion of females who ever had sex by age, following cohorts of women over the course of their lives from age 0-30 (blue lines).

**Data sources**: Angola 2015-16 (1); Benin 2017-18 (2); Burkina Faso 2010 (3); Burundi 2016-17 (4); Cameroon 2018 (5); Chad 2014-15 (6); Congo 2015-16; DRC 2013-14 (7); Ethiopia 2016 (8); Ghana 2014 (9); Guinea 2018 (10) Kenya 2014 (11); Madagascar 2021 (12); Malawi 2015-16 (13); Mali 2018 (14); Mozambique 2015 (15); Niger 2012 (16); Nigeria 2018 (17); Rwanda 2019-20 (18); Senegal 2019 (19); Sierra Leone 2019 (20); South Africa 2016 (21); Tanzania 2015-16 (22); Togo 2013-14 (23); Uganda 2016 (24); Zambia 2018 (25); Zimbabwe 2015 (26). Data on the share of girls/women ever having had sex by age was not available for Cote d’Ivoire, Somalia, Sudan, or South Sudan. For these 4 countries, we assumed that the distribution of ages of sexual debut was the same as for Ethiopia.

# Figure S3. Sexual mixing matrices


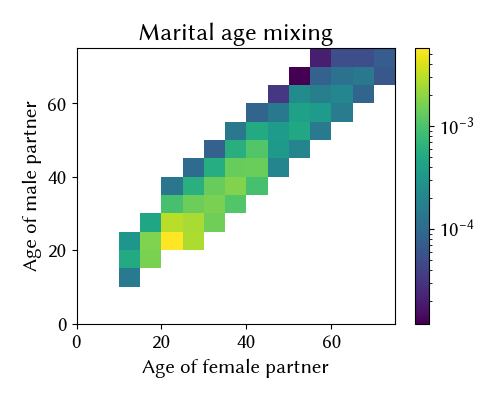


**Caption**: For each country, we input sexual mixing matrices that determine the probability of females of a given age selecting partners of each given age. The heatmap here represents a histogram showing the densities of simulated marital-type relationships between males and females by age for Angola (selected for illustrative purposes). In some DHS surveys, data are available on the proportion of females aged 15-19 who’ve had sex in the last 12 months with a partner 10+ years older than them, however we deemed this data to be subject to an unacceptable degree of reporting error and have chosen not to use it directly. Therefore, our sexual mixing matrices for each country are assumptions; in general, we assume that females are more likely to partner with males who are the same age or older than them, and less likely to partner with younger males.

# Figure S4. Age differences between partners


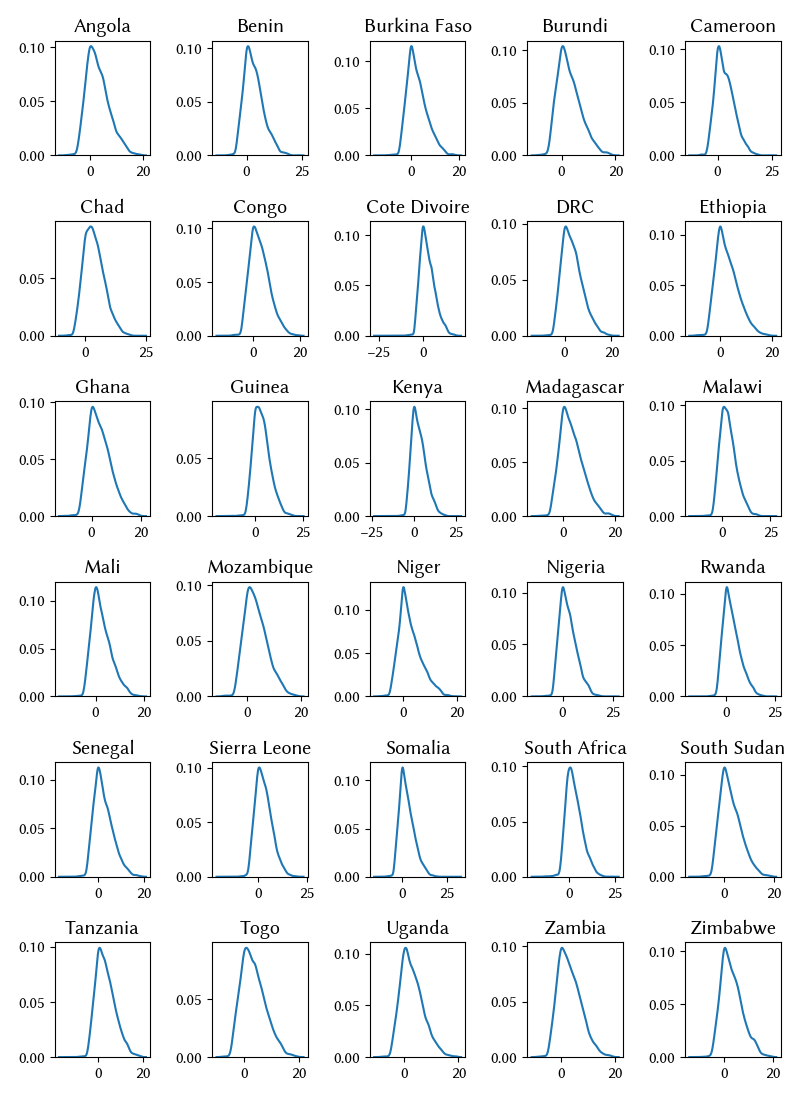


**Caption**: Densities of the simulated differences between the ages of male and female sexual partners.

# Figure S5. Share of females married by age


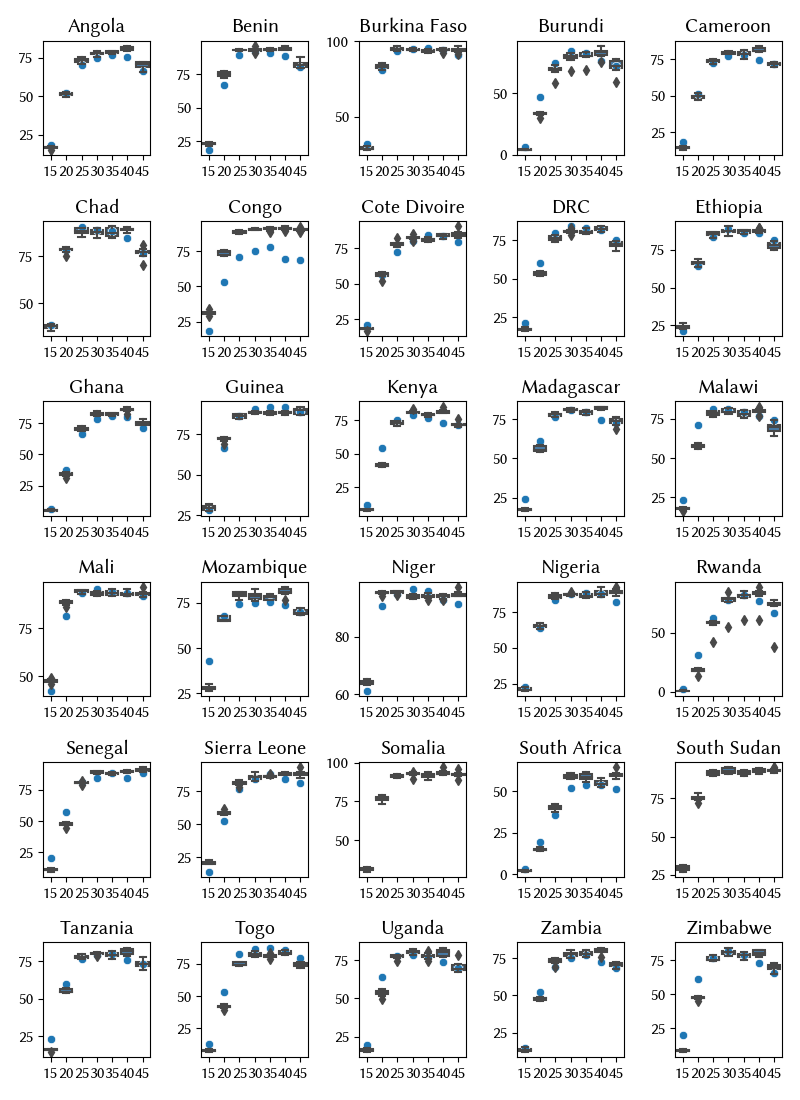


**Caption**: For each country, we collate data from DHS surveys (see sources below) on the proportion of girls/women married by age 15-19, 20-24, 25-29, 30-34, 35-39, 40-44, and 45-49 (blue dots). We then find marriage incidence rates for each country to match these data points. The box plots summarize the simulated proportion of females in marriage-like relationships every 5 years measured from 1985-2020.

**Data sources**: Angola 2015-16 (1); Benin 2017-18 (2); Burkina Faso 2010 (3); Burundi 2016-17 (4); Cameroon 2018 (5); Chad 2014-15 (6); Cote d’Ivoire 2011-12 (27); Congo 2015-16; DRC 2013-14 (7); Ethiopia 2016 (8); Ghana 2014 (9); Guinea 2018 (10) Kenya 2014 (11); Madagascar 2021 (12); Malawi 2015-16 (13); Mali 2018 (14); Mozambique 2015 (15); Niger 2012 (16); Nigeria 2018 (17); Rwanda 2019-20 (18); Senegal 2019 (19); Sierra Leone 2019 (20); South Africa 2016 (21); Tanzania 2015-16 (22); Togo 2013-14 (23); Uganda 2016 (24); Zambia 2018 (25); Zimbabwe 2015 (26). Data on the share of girls/women ever having had sex by age was not available for Somalia or South Sudan. For these 2 countries, we assumed that the proportions of females married by age was the same as for Ethiopia.

# Figure S6. Females with 1+ casual partner by age


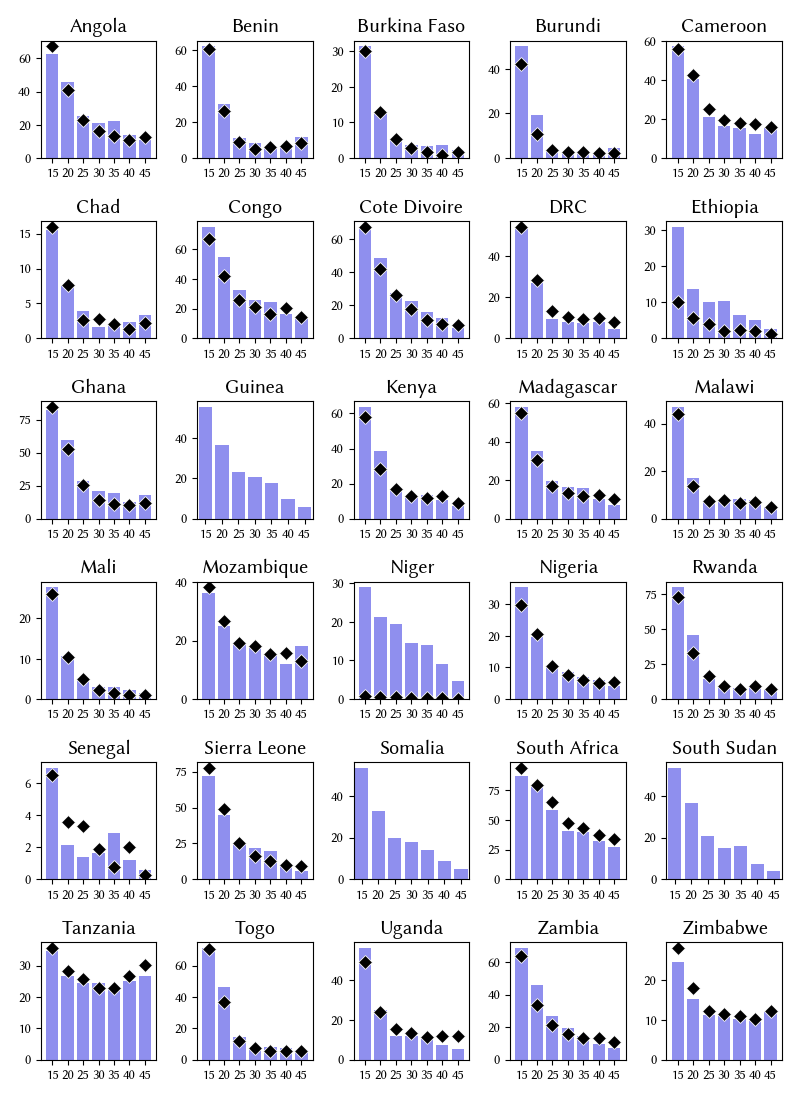


**Caption**: For each country, we plot the simulated percentages of sexually-active females aged 15-19, 20-24, 25-29, 30-34, 35-39, 40-44, and 45-49 who had 1+ casual partner over 2020 (blue bars). Alongside this, we plot data from DHS surveys on the percentage of women who reported sex with at least one non-marital, non-cohabiting partner in the last 12 months (black diamonds).

**Data sources**: Angola 2015-16 (1); Benin 2017-18 (2); Burkina Faso 2010 (3); Burundi 2016-17 (4); Cameroon 2018 (5); Chad 2014-15 (6); Cote d’Ivoire 2011-12 (27); DRC 2013-14 (7); Ethiopia 2016 (8); Ghana 2014 (9); Guinea 2018 (10) Kenya 2014 (11); Madagascar 2021 (12); Malawi 2015-16 (13); Mali 2018 (14); Mozambique 2015 (15); Niger 2012 (16); Nigeria 2018 (17); Rwanda 2019-20 (18); Senegal 2019 (19); Sierra Leone 2019 (20); South Africa 2016 (21); Sudan 1989-90 [(28)](https://www.zotero.org/google-docs/?broken=QYu8iG); Tanzania 2015-16 (22); Togo 2013-14 (23); Uganda 2016 (24); Zambia 2018 (25); Zimbabwe 2015 (26).

# Figure S7. Fit to cancers by age from unconstrained calibrations


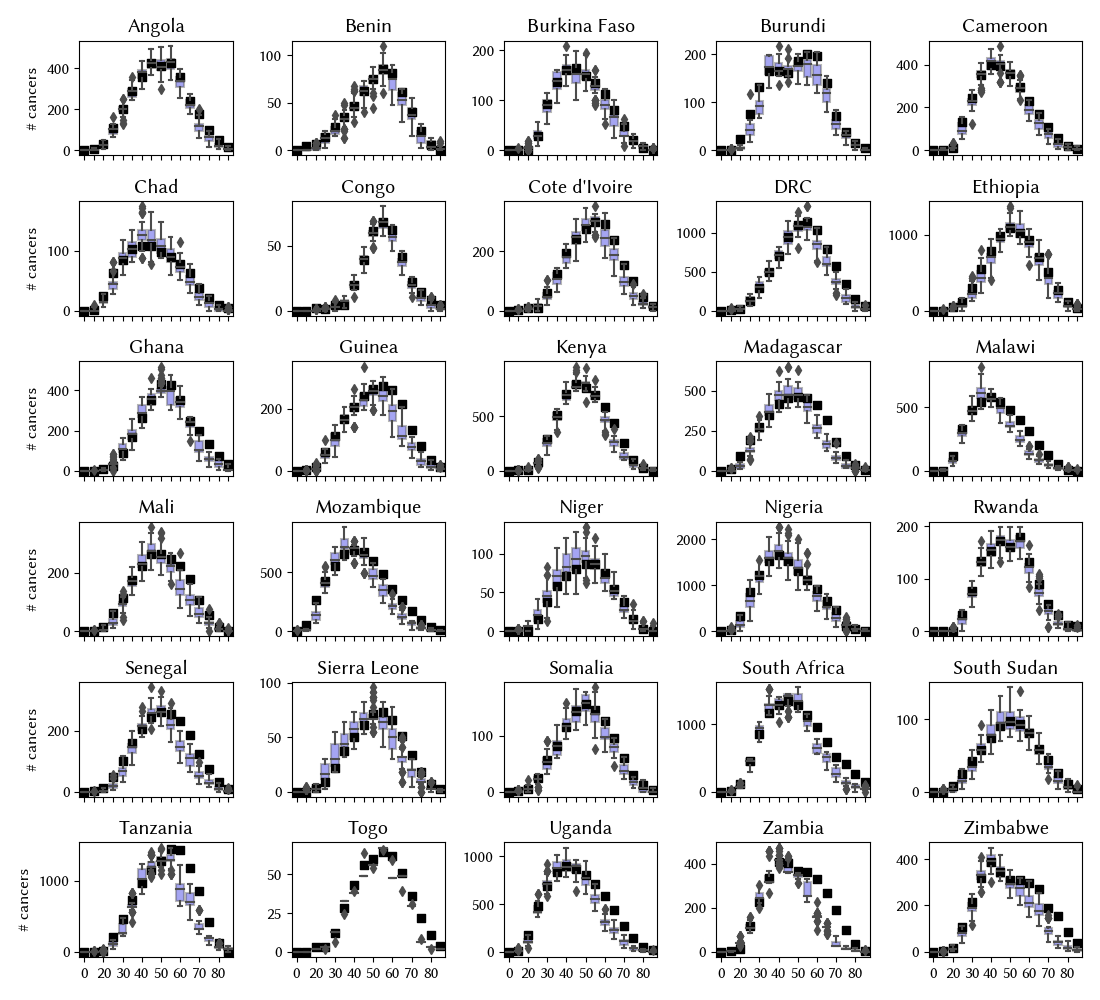


**Caption**: Model estimates generated from the top 50 best-fitting parameter sets from the unconstrained calibrations (box plots) along with data from GLOBOCAN on the distribution of cervical cancer by age.

# Figure S8. Fit to type distribution among invasive cervical cancers - constrained calibration


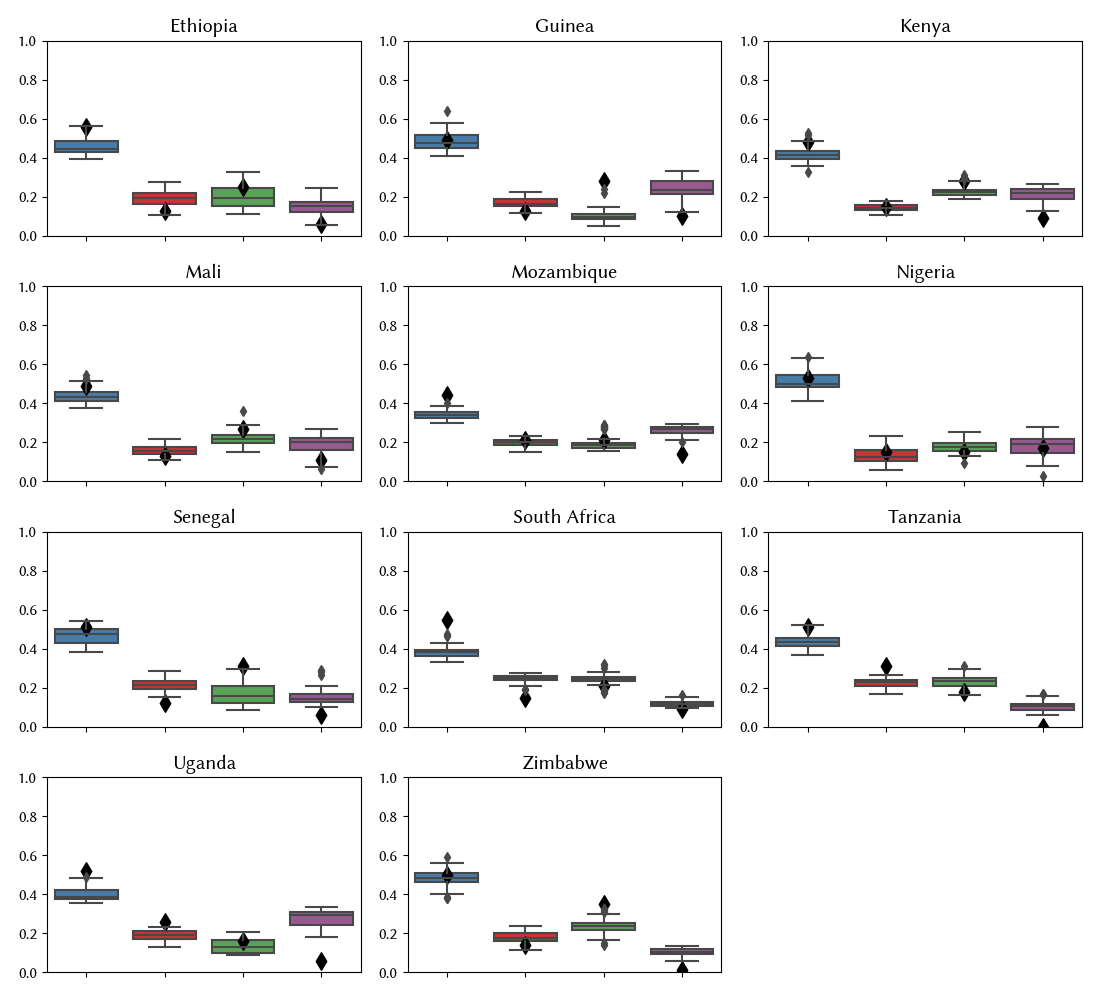


**Caption**: Model estimates of the distribution of HPV types found in women with invasive cervical cancer, generated from the top 50 best-fitting parameter sets from the constrained calibration.

# Figure S9. Fit to type distribution among invasive cervical cancers - immunovarying calibration


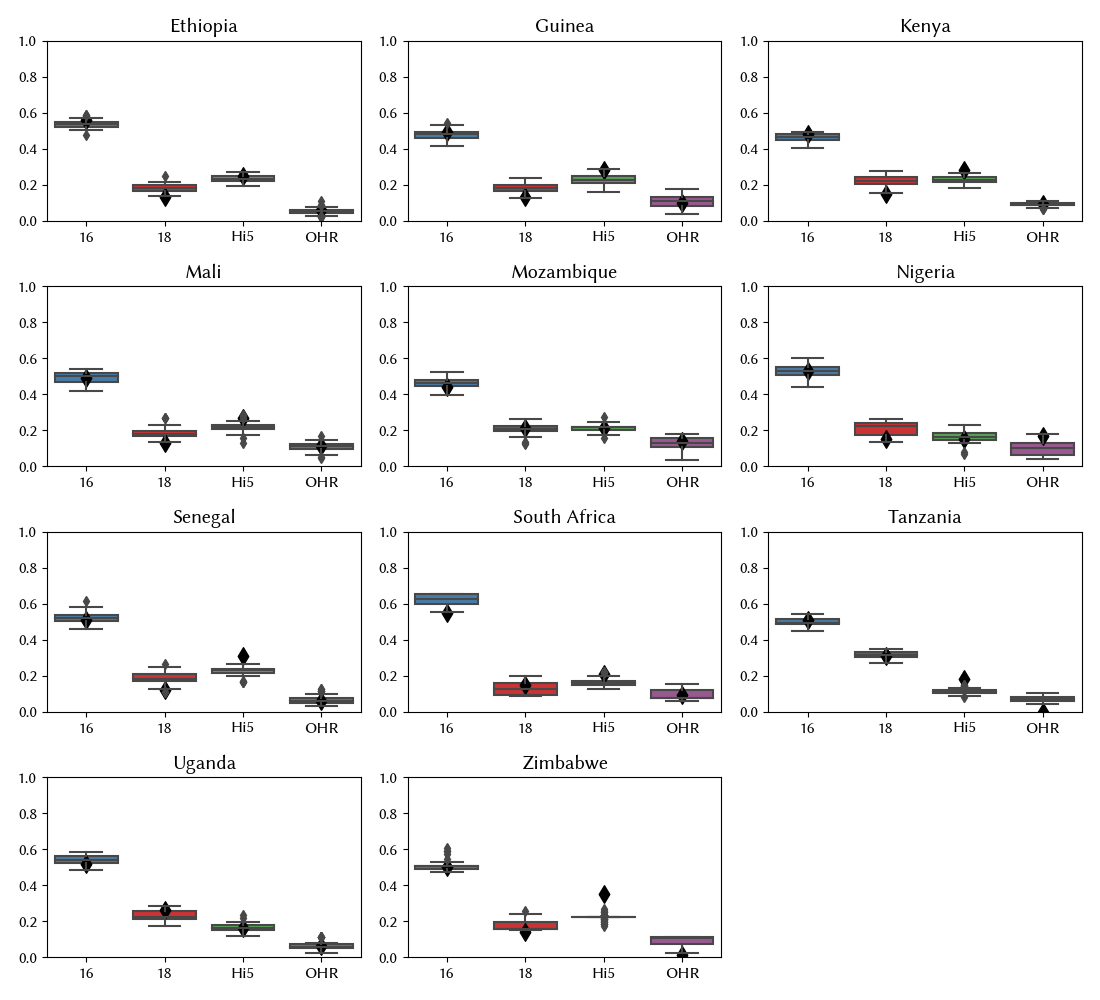


**Caption**: Model estimates of the distribution of HPV types found in women with invasive cervical cancer, generated from the top 50 best-fitting parameter sets from the constrained calibration.

# Figure S10. Fit to type distribution among invasive cervical cancers - unconstrained calibration


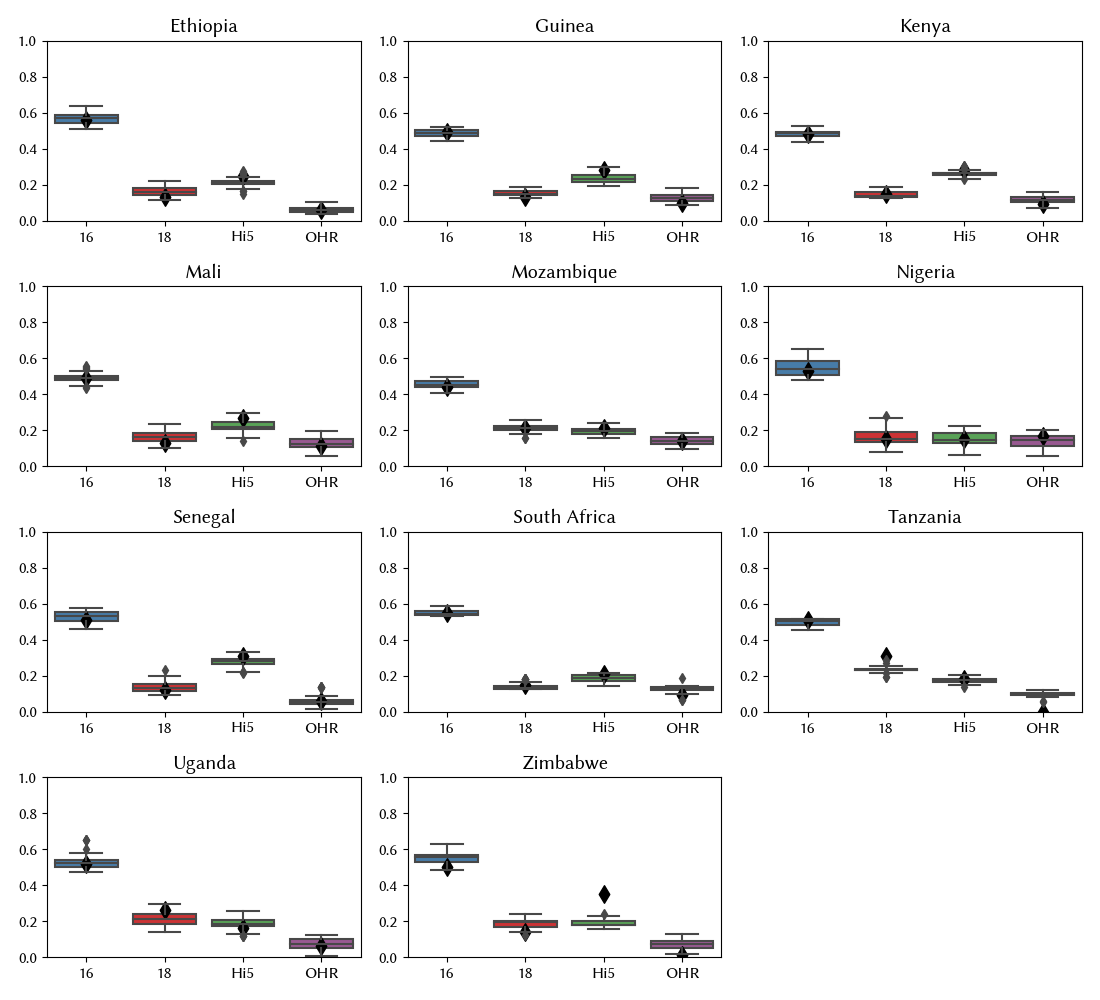


**Caption**: Model estimates of the distribution of HPV types found in women with invasive cervical cancer, generated from the top 50 best-fitting parameter sets from the unconstrained calibration.

# Figure S11. Posterior distributions – constrained calibration


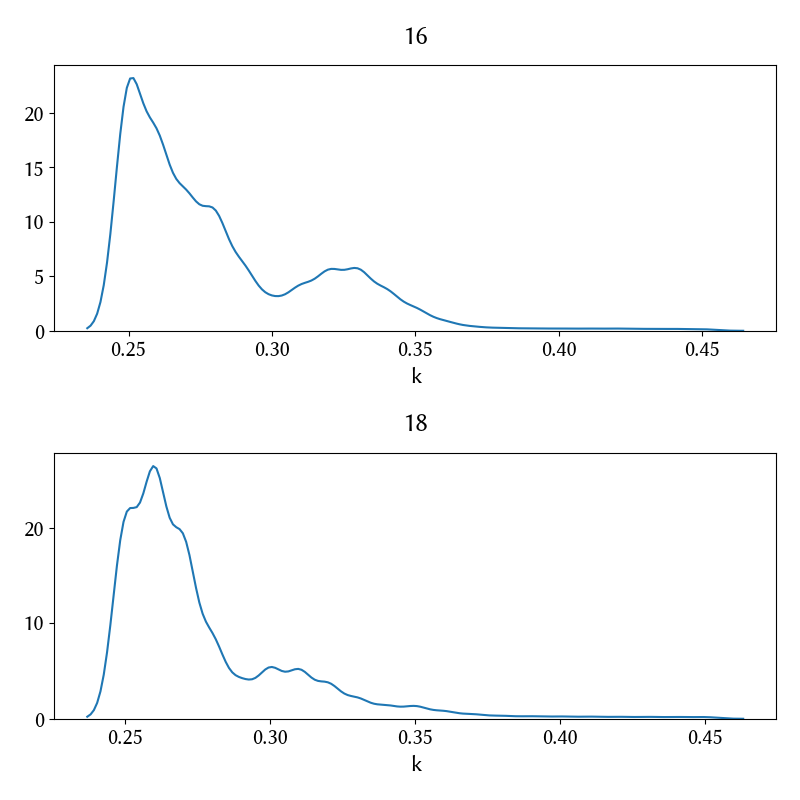


**Caption**: Posterior distributions of k_g_ from the constrained calibration.

# Figure S12. Posterior distributions – unconstrained calibration


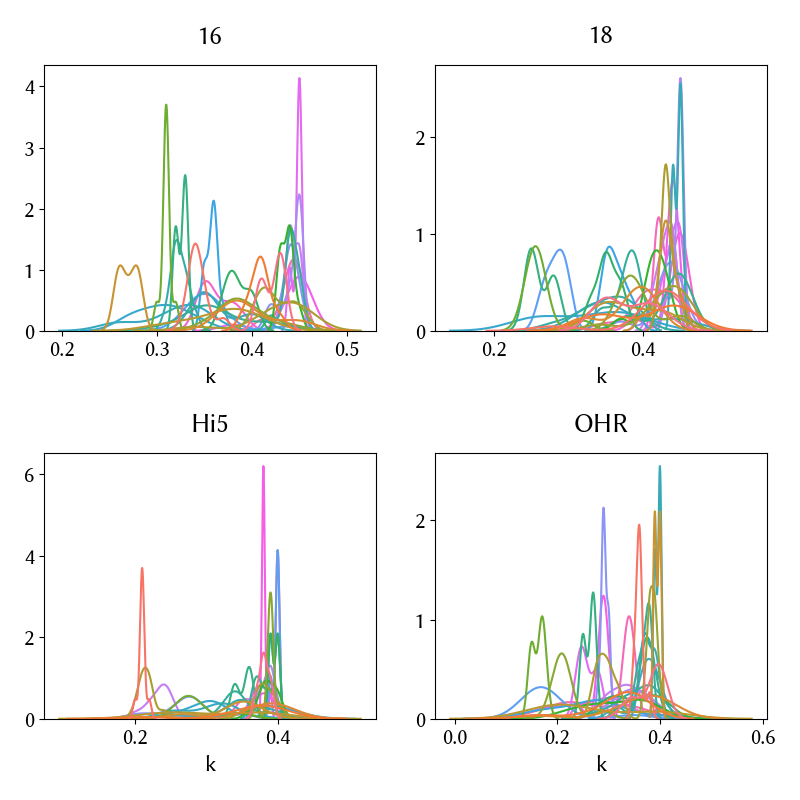


**Caption**: Posterior distributions of k_g_ for 30 countries from the unconstrained calibration. Each color represents a different country.

# References

1. Instituto Nacional de Estatística - INE/Angola, Minstério da Saúde - MINSA/Angola, ICF. Angola Inquérito de Indicadores Múltiplos e de Saúde (IIMS) 2015-2016 [Internet]. Luanda, Angola: INE, MINSA, and ICF; 2017. Available from: http://dhsprogram.com/pubs/pdf/FR327/FR327.pdf

2. Institut National de la Statistique et de l’Analyse Economique (INSAE), ICF. République Du Bénin Ciquième Enquête Démographique et de Santé au Bénin (EDSB-V) 2017-2018 [Internet]. Cotonou, Benin: INSAE/Benin and ICF; 2019. Available from: http://dhsprogram.com/pubs/pdf/FR350/FR350.pdf

3. Institut National de la Statistique et de la Démographie - INSD/Burkina Faso, ICF International. Burkina Faso Enquête Démographique et de Santé et à Indicateurs Multiples (EDSBF-MICS IV) 2010 [Internet]. Calverton, Maryland, USA: Institut National de la Statistique et de la Démographie - INSD/Burkina Faso and ICF International; 2012. Available from: http://dhsprogram.com/pubs/pdf/FR256/FR256.pdf

4. Ministère à la Présidence chargé de la Bonne Gouvernance et du Plan - MPBGP, Ministère de la Santé Publique et de la Lutte contre le Sida - MSPLS, Institut de Statistiques et d’Études Économiques du Burundi - ISTEEBU, ICF. Burundi Troisième Enquête Démographique et de Santé 2016-2017 [Internet]. Bujumbura, Burundi: MPBGP, MSPLS, ISTEEBU, and ICF; 2017. Available from: http://dhsprogram.com/pubs/pdf/FR335/FR335.pdf

5. Institut National de la Statistique/INS, ICF. République du Cameroun Enquête Démographique et de Santé 2018 [Internet]. Yaoundé, Cameroun: INS and ICF; 2020. Available from: https://www.dhsprogram.com/pubs/pdf/FR360/FR360.pdf

6. Institut National de la Statistique des Études Économiques et Démographiques - INSEED/Tchad, Ministère de la Santé Publique - MSP/Tchad, ICF International. Enquête Démographique et de Santé et à Indicateurs Multiples au Tchad (EDS-MICS) 2014-2015 [Internet]. 2016. Available from: http://dhsprogram.com/pubs/pdf/FR317/FR317.pdf

7. Ministère du Plan et Suivi de la Mise en œuvre de la Révolution de la Modernité - MPSMRM/Congo, Ministère de la Santé Publique - MSP/Congo, ICF International. République Démocratique du Congo Enquête Démographique et de Santé (EDS-RDC) 2013-2014 [Internet]. Rockville, Maryland, USA: MPSMRM, MSP, and ICF International; 2014. Available from: http://dhsprogram.com/pubs/pdf/FR300/FR300.pdf

8. Central Statistical Agency - CSA/Ethiopia, ICF. Ethiopia Demographic and Health Survey 2016 [Internet]. Addis Ababa, Ethiopia: CSA and ICF; 2017. Available from: http://dhsprogram.com/pubs/pdf/FR328/FR328.pdf

9. Ghana Statistical Service - GSS, Ghana Health Service - GHS, ICF International. Ghana Demographic and Health Survey 2014 [Internet]. Rockville, Maryland, USA: GSS, GHS, and ICF International; 2015. Available from: http://dhsprogram.com/pubs/pdf/FR307/FR307.pdf

10. Institut National de la Statistique, ICF. Guinea Demographic and Health Survey (EDS V) 2016-18 [Internet]. Conakry, Guinea: INS/Guinea and ICF; 2019. Available from: http://dhsprogram.com/pubs/pdf/FR353/FR353.pdf

11. Kenya National Bureau of Statistics, Ministry of Health/Kenya, National AIDS Control Council/Kenya, Kenya Medical Research Institute, National Council for Population and Development/Kenya. Kenya Demographic and Health Survey 2014 [Internet]. Rockville, MD, USA; 2015. Available from: http://dhsprogram.com/pubs/pdf/FR308/FR308.pdf

12. Institut National de la Statistique (INSTAT), ICF. Enquête démographique et de santé à Madagascar (EDSMD-V) 2021 [Internet]. Antananarivo, Madagascar et Rockville, Maryland, USA: INSTAT, ICF; 2022. Available from: https://www.dhsprogram.com/pubs/pdf/FR376/FR376.pdf

13. National Statistical Office/Malawi, ICF. Malawi Demographic and Health Survey 2015-16 [Internet]. Zomba, Malawi: National Statistical Office and ICF; 2017. Available from: http://dhsprogram.com/pubs/pdf/FR319/FR319.pdf

14. Institut National de la Statistique - INSTAT, Cellule de Planification et de Statistique Secteur Santé-Développement, ICF. Mali Demographic and Health Survey 2018 [Internet]. Bamako, Mali: INSTAT/CPS/SS-DS-PF and ICF; 2019. Available from: http://dhsprogram.com/pubs/pdf/FR358/FR358.pdf

15. Ministério da Saúde- MISAU, Instituto Nacional de Estatística - INE, ICF. Inquérito de Indicadores de Imunização, Malária e HIV/SIDA em Moçambique (IMASIDA) 2015 [Internet]. Maputo/Moçambique: MISAU/Moçambique, INE, and ICF; 2018. Available from: http://dhsprogram.com/pubs/pdf/AIS12/AIS12.pdf

16. Institut National de la Statistique - INS/Niger, ICF International. Niger Enquête Démographique et de Santé et à Indicateurs Multiples (EDSN-MICS IV) 2012 [Internet]. Calverton, Maryland, USA: INS/Niger and ICF International; 2013. Available from: http://dhsprogram.com/pubs/pdf/FR277/FR277.pdf

17. National Population Commission - NPC, ICF. Nigeria Demographic and Health Survey 2018 - Final Report [Internet]. Abuja, Nigeria: NPC and ICF; 2019. Available from: http://dhsprogram.com/pubs/pdf/FR359/FR359.pdf

18. National Institute of Statistics of Rwanda - NISR, Ministry of Health - MOH, ICF. Rwanda demographic and health survey 2019-20 [Internet]. Kigali, Rwanda and Rockville, Maryland, USA: NISR/MOH/ICF; 2021. Available from: https://www.dhsprogram.com/pubs/pdf/FR370/FR370.pdf

19. Agence Nationale de la Statistique et de la Démographie (ANSD), ICF. Senegal: Enquête Démographique et de Santé Continue (EDS- Continue) 2019 [Internet]. Dakar/ Sénégal: ANSD/ICF; 2020. Available from: https://www.dhsprogram.com/pubs/pdf/FR368/FR368.pdf

20. Statistics Sierra Leone - StatsSL, ICF. Sierra Leone Demographic and Health Survey 2019 [Internet]. Freetown/Sierra Leone: StatsSL/ICF; 2020. Available from: https://www.dhsprogram.com/pubs/pdf/FR365/FR365.pdf

21. National Department of Health, ICF. South Africa Demographic and Health Survey 2016 [Internet]. Pretoria: National Department of Health - NDoH - ICF; 2019. Available from: http://dhsprogram.com/pubs/pdf/FR337/FR337.pdf

22. Ministry of Health CD, Ministry of Health - MoH/Zanzibar, National Bureau of Statistics - NBS/Tanzania, Office of Chief Government Statistician - OCGS/Zanzibar, ICF. Tanzania Demographic and Health Survey and Malaria Indicator Survey 2015-2016 [Internet]. Dar es Salaam, Tanzania: MoHCDGEC, MoH, NBS, OCGS, and ICF; 2016. Available from: http://dhsprogram.com/pubs/pdf/FR321/FR321.pdf

23. Ministère de la Planification, du Développement et de l’Aménagement du Territoire - MPDAT/Togo, Ministère de la Santé - MS/Togo, ICF International. Togo Enquête Démographique et de Santé 2013-2014 [Internet]. Rockville,Maryland, USA: MPDAT/Togo, MS/Togo and ICF International; 2015. Available from: http://dhsprogram.com/pubs/pdf/FR301/FR301.pdf

24. Uganda Bureau of Statistics - UBOS, ICF. Uganda Demographic and Health Survey 2016 [Internet]. Kampala, Uganda: UBOS and ICF; 2018. Available from: http://dhsprogram.com/pubs/pdf/FR333/FR333.pdf

25. Zambia Statistics Agency - ZSA, Ministry of Health - MOH, University Teaching Hospital Virology Laboratory - UTH-VL, ICF. Zambia Demographic and Health Survey 2018 [Internet]. Lusaka, Zambia: ZSA, MOH, UTH-VL and ICF; 2020. Available from: https://www.dhsprogram.com/pubs/pdf/FR361/FR361.pdf

26. Zimbabwe National Statistics Agency, ICF International. Zimbabwe Demographic and Health Survey 2015: Final Report [Internet]. Rockville, Maryland, USA: Zimbabwe National Statistics Agency (ZIMSTAT) and ICF International; 2016. Available from: http://dhsprogram.com/pubs/pdf/FR322/FR322.pdf

27. Institut National de la Statistique - INS/Côte d’Ivoire, ICF International. Côte d’Ivoire Enquête Démographique et de Santé et à Indicateurs Multiples 2011-2012 [Internet]. Calverton, Maryland, USA: INS/Côte d’Ivoire and ICF International; 2013. Available from: http://dhsprogram.com/pubs/pdf/FR272/FR272.pdf

28. Department of Statistics at the Ministry of Economic and National Planning/Sudan, Institute for Resource Development/Macro International. Sudan Demographic and Health Survey 1989/1990 [Internet]. Columbia, Maryland, USA: Institute for Resource Development/Macro International; 1991. Available from: http://dhsprogram.com/pubs/pdf/FR36/FR36.pdf
